# Why a responsibility sensitive healthcare system is not disrespectful

**DOI:** 10.1007/s11019-025-10262-x

**Published:** 2025-03-14

**Authors:** Lydia Tsiakiri

**Affiliations:** Department of Political Science and the Centre for the Experimental-Philosophical Study of Discrimination (CEPDISC), Aarhus University, Aarhus, Denmark

**Keywords:** Luck egalitarianism, (Dis)respect, Doctrine of double effect, Kant, Duties

## Abstract

The prevalence of non-communicable diseases, the related increased medical costs, and the recent public health emergency bring out more forcefully pre-existing dilemmas of distributive justice in the healthcare context. Under this reality, would it be justified to hold people responsible for their taken lifestyle decisions, or would it constitute an instance of unjustified disrespectful treatment? From a respect-based standpoint, one could argue that a responsibility-sensitive healthcare system morally disrespects the imprudent ones engaging in disadvantageous differential treatment to their detriment. In contrast, however, we might also have luck egalitarian reasons that explain why this differential treatment is not unjust. Luck egalitarianism is a responsibility-sensitive theory of distributive justice, which argues that it is bad if some people are worse off than others through no voluntary fault of their own. In this paper, I clarify the concerns about disrespect raised against the luck egalitarian viewpoint and offer possible respect-based reasons for why this might not be the case grounded in deontological concepts. First, I employ a revised Double-effect case to support responsibility-sensitive rationing. In the last part of the paper, these are further supported through the Kantian Formula of Humanity supplemented by the concept of duties.

## Introduction

During the last 50 years, our world has witnessed a changing pattern of disease. The acute infectious and deficiency illnesses that used to determine the burden of disease have been marginalized by the prevalence of non-communicable diseases (NCDs), such as diabetes, cancer, and heart disease. The 2013 Global Burden of Disease Study (Lim et al. 2013) suggested the current predominance of NCD risks highlighting the substantial shift between 1990 and 2010, from risk factors associated with communicable diseases (CDs) in children to those associated with NCDs in adults. The same study undertaken again in 2019 confirmed this shift. NCDs were now among the top causes of all disease burden in adults, while those and self-inflicted injuries amounted to more than half of the total disease burden for 11 countries (Vos et al. 2019).

These changing risk and disease patterns also matter for the costs incurred by private health insurance and public healthcare systems (Rasmussen et al. 2004; Long et al. 2006; Golan 2010). In 2021, for instance, the healthcare expenditure for type 2 diabetes treatment across Europe was €176 billion (International Diabetes Federation 2023), while in 2018, the EU spent almost €97 billion for cancer (Hofmarcher et al. 2020). This apparent medical and financial pressure casts light on key questions related to the justified distribution of these costs of treatment and the extent to which individual behavior should be taken into account when the rationing of scarce healthcare resources is required (Cappelen and Norheim 2005; Golan 2010; Sharkey, and Gillam 2010; Albertsen 2020).

In connection with these, during the last 20 years, studies in the UK and the US have shown that personal responsibility plays an important role in the public’s thinking about health, while it urges for greater personal accountability in the healthcare context (Wellings 2017; Holt-White 2019). Similarly, an international survey on the values of health professionals concerning healthcare-spending priorities classified the spending on cancer treatment for smokers as the least important (Salkeld et al. 2007). This could imply an important role for individual responsibility. Reflecting the above, a continuously increasing number of policies has introduced various incentives to encourage individuals’ responsible behaviour, with countries like the UK, Germany, and Sweden constituting the most frequently mentioned examples of countries implementing such legislation (Voigt 2011; Traina and Feiring 2020). In the British clinical settings, for instance, one might be asked to engage in lifestyle changes, such as quitting smoking or losing weight, to qualify for treatment (Pillutla et al. 2018). Building on all these, the justifiability of personal responsibility considerations in healthcare seems more urgent than ever. This article contributes to the ongoing discussion by theoretically assessing whether responsibility-sensitive healthcare policies engage in disrespectful treatment, as critics would hold (Anderson 1999; Wolff 1998; 2010; Cavallero 2011; Ahola-Launonen 2018; Kennett, 2024).

The paper proceeds as follows. In the next section, I present the fairness-based arguments in favour of personal responsibility for health. These are based on the luck egalitarian theory of distributive justice. Such arguments for personal responsibility considerations in the context of health have often been met by respect-based critiques. I present some of these and then, as the paper’s main contribution, I provide a respect-based reasoning grounded in the ‘double effect doctrine’ and the Kantian ‘formula of humanity,’ advocating for a qualified yes regarding the consideration of personal responsibility for health.

## Luck egalitarianism (in health)

Imagine that Jack and John, colleagues at the same office with the same salary, are both 60 years old and equally capable of performing. Jack decides to work overtime to increase his income, while John chooses not to do so. The consequent inequality would intuitively be considered just because it reflects differential exercises of responsibility.

But would a similar intuition also be the case regarding their health status if Jack had chosen a healthy lifestyle, whereas John had a neutral or an unhealthy one? To respond to this question, let me reform the initial example. In this case, Jack chose to follow a healthy diet and exercise five times weekly throughout his adult life. Today, despite being 60 years old, he is healthy and has never needed medical care. On the contrary, although John adopted an equally healthy diet and exercised as much as Jack did, he has also been a heavy smoker for the last 20 years with relatively deteriorated health. Would these chosen lifestyles justify John’s deprioritization or obligation to pay higher out-of-pocket contributions for treatment when both patients simultaneously arrive sick at an overwhelmed hospital? In contrast to the work example, I think that our intuition could not provide a unanimous response to the above question. For some, applying a responsibility-sensitive approach to this area would be morally objectionable due to the basic and inalienable value of health conceived as an undeniable right owed equally to everyone (Andersen et al. 2013; Eyal 2013). For others, however, such treatment would seem able to ensure distributive justice in the healthcare system.

The submitted reasons for why the inequality between Jack and John is not unjust reflect the sentiments endorsed by luck egalitarianism.^1^ This is a responsibility-sensitive theory of distributive justice, which argues that ‘it is bad if some people are worse off than others through no voluntary fault or choice of their own’ (Parfit 1984, p. 3, n. 5) aiming exclusively at remedying inequalities of pure luck (Dworkin 1981; Arneson 1989; Cohen 1989; Roemer 1993; Rakowski 1993; Knight 2013; Lippert-Rasmussen 2016). However, how does this abstract theory fare when applied to real-world contexts, as in the case of the healthcare system? Luck egalitarianism responds by arguing that inequalities in health expectancy attributable to personal priorities are justified and might not be compensated (Segall 2010), recognizing, nevertheless, the potentially determinant effect of circumstances on one’s ‘personal priorities’ (Segall 2010; Albertsen 2015).

Yet, the employment of this still broad view in the current discussion requires further clarification. Who is the responsible I am talking about here? What is the source of her responsibility, and what are the actual embodiments of this responsibility-sensitive approach regarding healthcare provision that I am interested in? Commencing with the first one, I stand by luck egalitarians’ explicit interest in all the kinds of imprudent behaviour able to deteriorate one’s health and not exclusively in those for which society tends to hold people responsible, as it is obesity, smoking, and alcoholism. In addition, I classify as responsible for their worse-off condition those whose choices seem to have directly put them in that condition, setting aside those whose choices’ expected value could have disadvantageously affected them, but it did not eventually do so.

Having clarified this, one could also ask: Responsibility for what? Here, I must state my exclusive interest in responsibility for prognosis. Furthermore, contemporary literature has brought into the spotlight two antagonistic models for approaching responsibility, the backward- and the forward-looking ones. Under the former, an individual’s responsibility is assessed based on whether they have already contributed to the creation of the distributed burdens and benefits of the current state of affairs, whereas under the latter, individuals are held responsible on account of intended future outcomes (Cappelen and Norheim 2005; Feiring 2008; Nielsen and Andersen 2014). In the context of this article, I adopt the former concept, since it is compatible with my interest in responsibility for prognosis.

Lastly, regarding actual embodiments of this broad view, to wit, regarding responsibility-sensitive policies, I do not consider cases of complete abandonment. Everyone’s basic needs for health and healthcare should be respected and discussions regarding a responsibility-sensitive resource allocation should start above that threshold (Segall 2010). Therefore, I advocate for more moderate responsibility-sensitive policies (Voigt 2011). In other words, I argue in favour of measures such as the imposition of longer waiting times before the provision of treatment under non-life-threatening conditions, or higher insurance co-payments and premiums for receiving treatment (Davies et. al. 2024; Cappelen and Norheim 2005; Voigt 2007; Daniels 2011; Cavallero 2011; Albertsen 2020).

## The critical side of the coin

The responsibility-sensitive approach to healthcare, however, has been criticized on multiple grounds and from different starting points. Among the long list of critiques, the more often cited ones are the criticism grounded in the blurry choice-circumstances dichotomy (Sheffler 2005; Bognar 2019), which potentially impedes any clear causality attribution,^2^ a concern for over-individualization, and the respect-based family of objections; the harshness, intrusiveness, and, more recently, the discrimination critique.

## Responsibility-scepticism and over-individualization

The first criticism often raised against the above presented approach pertains to a scepticism about our ability to accurately identify responsibility. Unhealthy behaviour is statistically more widespread among those with lower socioeconomic status (Capellen and Norheim 2005; Feiring 2008; Pillutla et al. 2018). Since our choices are considerably affected by social circumstances (Ahola-Launonen 2015; 2018; Feiring 2008; Barry 2008; Albertsen 2015, 2020; Friesen 2016; Traina and Feiring 2020), and people do not live in a vacuum (Minkler 1999), but in a multifactorial world of environmental, societal, and genetic elements that co-define causality (Ahola-Launonen 2015; 2018; Buyx 2008; Albertsen 2015; Levy 2019), it seems questionable whether a clear line between choice and circumstance is even plausible to be drawn, let alone to ground on this basis any further principled discussion. However, even though this constitutes a critique worthy of consideration, it remains on the margins of the current argumentation given that it pertains to a different discussion than the one I aim to address here. In essence, whenever the issue of the healthcare system’s responsibilization is put on the table, at least, two distinct questions attract scholars’ attention; a principled and a practical one. The former—the one I also contribute to here—asks whether we *should* consider personal responsibility when we look for distributive justice; the latter whether we *can* identify who exactly is responsible for what. According to my understanding, which also builds on several scholars’ engagement with the principled discussion prior to providing an explicit answer to the practical one (Dworkin 1981; Arneson 1989; Cohen 1989; Roemer 1993; Rakowski 1993; Knight 2013; Lippert-Rasmussen 2016; Albertsen 2020), we need a convincing affirmative response to the former before we start reflecting on the practicalities. But still, even if things work the other way around, unless a fixed answer is reached under the practical discussion able to rule out with definite certainty any possibility for detecting the truly responsible now and/or in the distant future, we ought to assess the moral justifiability of the system’s responsibilization for the just treatment of the currently and potentially identifiable prudent and imprudent.

Other critics question the validity of the individualistic, as they call it, interpretation of the theory expressing a concern for over-individualization. They claim that by emphasizing the demandingness of attributing responsibility and how unstable foundation for distributive justice this could be, luck egalitarianism’s ultimate, but misdiagnosed even from its proponents, aim has always been to redirect our focus to non-individualistic understandings (Ahola-Launonen 2018). This paper does not take sides on the issue. Nonetheless, as the rest of this section suggests, luck egalitarianism has been mainly criticized for its alleged disrespectful treatment of the responsible. By recognizing the prevalence of this family of critiques, what I adopt here is a perspective more attuned to the individualistic interpretation of the theory not, as I mentioned, to provide a response to the ongoing discussion about its ultimate aim, but, instead, to respond to the objections raised by many against it.

## Respect-based objections: harshness, intrusiveness and discrimination

Moving now to the respect-based family of objections the most famous one among them is the harshness objection. It concerns the victims of bad option luck and the alleged luck egalitarian disrespect towards them. Fleurbaey (1995) and Anderson (1999) argue that luck egalitarianism treats those who are responsible for their abject condition with counterintuitive harshness. In fact, it lets them bear the disproportional costs of their choices regardless of how high they may be, providing too little (or nothing at all) for the restoration of their health and acting inconsistently with its promises for equal respect for everyone.

Luck egalitarians have provided various replies to this. Some suggest, for instance, that more pluralist versions of luck egalitarianism provide an adequate response, as other values than fairness are taken into account, leading to more moderate responsibility-sensitive policies (Voigt 2011). Others, like Segall (2010), advocate for people’s fundamental right to basic and sufficient health and healthcare before engaging in any discussions on responsibility-sensitive resource allocation. Yet while the luck egalitarian responses pointing to such institutional arrangements might avoid the charges for counterintuitive harshness and disproportional costs for the imprudent, it is less clear that they avoid disrespect. In other words, although this is not a discussion about extreme measures, deprioritisation of any form seems almost equally able to constitute a bearer of disrespect (De Marco 2024). Consequently, despite all the luck egalitarian efforts to resist this criticism, the concern for unequal recognition of respect to the others’ person remains.

In a similar vein, Wolff (1998; 2010) has proposed another worth considering objection; the intrusiveness critique. Following him, the implementation of luck egalitarian institutions and responsibility-sensitive policies entails an unjustified invasion to people’s private sphere. The core idea of this concern comes from the anticipated process of gaining access and collecting the required data to detect the truly responsible and the feelings of distrust and shame that this intrusive investigation might cause. Under a similar argumentative line, Preda and Voigt (2023) denounce luck egalitarian institutions for failing to treat potential claimants with sufficient politeness and due respect demanding sometimes shameful revelations at the altar of collecting those data needed to determine whether someone is truly blameworthy. Luck egalitarians have attempted to debunk this line of criticism invoking once again pluralist, sufficientarian, and prioritarian versions of luck egalitarianism. According to a pluralist understanding, for instance, when assessing the justifiability and effectiveness of these policies, we should also consider their impact on the overall welfare of those directly affected by them (Knight 2009), while according to a sufficientarian or a prioritarian understanding, any kind of shameful revelation might not be even needed (Albertsen, and Knight 2015). Yet, the system’s reasonable desire to be certain about one’s responsibility before we impose on them any kind of disadvantageous differential treatment seems to question the possibility of completely refuting the stated concern and the implied disrespect.

Finally, scholars, who currently reflect on responsibility-sensitive healthcare policies, often underline the potentially discriminatory underpinnings of them (Anderson 1999; Johann 2021; Björk 2021; Kennett, 2024). For instance, Pillutla et al. (2018) considers that in relation to smokers and obese patients’ access to elective surgery only after they have quit smoking for two months or lost some weight, respectively, while Rebecca Brown (2019) when assesses the justifiability of excluding obese women from publicly funded fertility treatments, like in vitro fertilization (IVF).^3^ In this context, given that respect for the commitment to everyone’s equal moral worth is found at the core of both relational egalitarianism and respect-based wrongful discrimination (Hellman 2008), the latter is perceived as potentially able to provide a new angle to the ongoing discussion refreshing the already raised respect-based concerns against luck egalitarianism (Albertsen, and Tsiakiri 2023; Albertsen 2024). For instance, the mental-state account of disrespectful discrimination notes, in brief, that the wrongfulness of discrimination is detected in the morally objectionable mental states of the discriminator regarding the inferior moral worth of the discriminatee (Alexander 1992; Lippert-Rasmussen 2013). Interpreting luck egalitarianism and its applications under such a viewpoint would potentially mean that the responsibilization of the medical system is seen as built on a problematic mental state about the inferior moral worth of the imprudent that justifies the implementation of disadvantageous differential treatment to their detriment. In contrast to the previous cases, since this critique started only recently being discussed (Albertsen, and Tsiakiri 2023; Albertsen 2024), with many even questioning its validity, luck egalitarians have not attempted to overcome it to a considerable extent, yet.

Summing all these up, there are some reasons to think that a responsibility-sensitive healthcare system might lead to a disrespectful treatment of the imprudent. In the rest of this article, however, I will present arguments upon which basis we may, contrary to such claims, have respect-based reasons to introduce responsibility-sensitive policies. These arguments invoke two concepts of the deontological tradition—i.e. the double effect doctrine and the Kantian duties account intertwined with the categorical Formula of Humanity—suggesting that a responsibility-sensitive healthcare system can constitute a guarantor and promoter of respectful interaction between itself and the citizens.

## Disrespectful differential treatment or ‘double effect’? A trolley problem analysis

In 1967, Philippa Foot attempted to advocate for the permissibility^4^ of certain harms grounded in the significance of the agent’s intentions by introducing the following thought experiment. In its initial scenario, the so-called ‘Trolley problem’ refers to the driver of a runaway trolley on an imaginary railway line. The trolley is on a lethal collision course with five workers, and the driver can only divert its course onto another track, where there is only one worker. If the trolley continues undisturbed on its course, five people will die. Although the driver did not intend to bring about this outcome, would it not be more ethically justified to divert the trolley and kill the one to save the five, causing the least possible suffering? The double effect doctrine provides an important perspective on this dilemma. According to the latter, it is morally permissible to cause some unintended but foreseeable harm in one’s effort to bring about some good, provided that certain conditions are met (Jackson 2011; McIntyre 2019).

As just stated, the doctrine comes with a set of conditions that, if met, would justify diverting the trolley towards the lone worker. First and foremost, the action in question must satisfy the condition of proportionality. The diversion of the trolley is regarded as justified when the intended positive moral value of the action surpasses the foreseen negative moral value of its side effect (Jackson 2011; McIntyre 2019). Yet the action should also satisfy the harm minimization condition. The agent must aim to cause the least possible suffering, even if this implies an additional risk for herself or the loss of some of the intended benefit (Walzer 1977). Adding to those, the discussed action must be difficultly preventable and morally acceptable, or otherwise, an action that in its very essence is good or at least indifferent. Lastly, it should be clear that the foreseen morally considerable harm constitutes exclusively a side effect and not a means of pursuing a different good end (Badger 2011; Jackson 2011; Woollard and Howard-Snyder 2022; McIntyre 2019). Building on the above, provided that the double effect doctrine and its conditions advise us in favour of saving the greatest number of people, a consequentialist underpinning seems to be present. But what if we had a one-to-one scenario? Would the doctrine of double effect be able to suggest a justified response to that case? What if the aspect of personal responsibility for the worker’s condition was also present? Would that make any difference? And eventually could this thought experiment be revised and applied in the current discussion of the consideration of personal responsibility in the healthcare context providing us with a reply regarding the justifiability of a responsibility-sensitive healthcare system?

In the one-to-one reformed scenario, the trolley is on a lethal collision course with one prudent worker who is on the railway lines only because he stumbled (~ bad brute luck). The driver can only divert the trolley’s course onto another track, where another worker is found. The latter, despite being informed of the possible train passing that very day and time decided to go picking flowers around the railway lines choosing ‘grossly reckless conduct’ (Arneson 2000, 348) and, consequently, exposing himself to a probable threat (~ bad option luck). Even though in this case, by saving either the prudent or the imprudent worker we save one and sacrifice as a side effect also one, we might have some responsibility-sensitive reasons to save the least deserving of the danger they face without, however, disrespecting any of the parties concerned here.

Under the healthcare discussion, the trolley problem could be invoked in the following form. Instead of one trolley that threatens to kill the prudent or the imprudent, one could suggest that under conditions of scarcity, the trolley could be replaced by one available cornea transplant and the two workers by two patients assessed on the basis of responsibility for prognosis. Patient no. 1 has always been taking care of their eyes. Yet, despite that, they have recently suffered a bacterial infection which, regardless of the patient’s immediate seeking for medical help, has led to a disruption of the normal functioning of their cornea. In contrast, patient no. 2 has always been an obsessive model maker in their free time systematically using power tools without wearing safety glasses and exposing, as a result, their eyes to dangerous flashes of light. On top of that, they also used to overusing the same contact lenses deteriorating considerably their prognosis for needing a cornea transplant and a more heavily invasive surgery. What should the doctors do regarding the allocation of this scarce medical resource, and could the double effect doctrine be of some help here? Could somebody suggest that, in this case, it is more ethically justified to deprioritize the imprudent one intending to alleviate the prudent?

To respond to the above question let me examine the health scenario’s compatibility with the double effect doctrine and its conditions. First and foremost, it must be stated explicitly that the medical system’s intention is to help/alleviate the prudent patient and not to deprioritize the imprudent. Simply put, its intention is to treat and protect the one who cannot escape from his bad brute luck despite having done his best to do so; the deprioritization of the imprudent individual is merely a foreseen but not an intended side effect. Building on this, what about the first two conditions of proportionality and harm minimization? Could they be fulfilled when the two patients are equal except for the aspect of responsibility for prognosis? My initial assumption would be that our choice to prioritize either the prudent or the imprudent one would not make any difference regarding proportionality and harm minimization. Yet, I must suggest that the condition of proportionality seems eventually to be indirectly met. This is because prioritizing the prudent, and, at the exact same moment, deprioritizing the imprudent on the basis of his poor health/eye-related choices and habits, one could produce a greater positive moral value in the long term because it incentivizes a healthier lifestyle, and under the current subcase, caring for the health of one’s eyes (Davies et. al. 2024; Callahan 2013). Relevant empirical evidence has shown that the implementation of policies that either directly or subtly reward people with healthy lifestyles, while disapproving, demoralizing and even stigmatizing people who engage in unhealthy actions, behaviours, and habits nudges the general population into the adoption of a healthier lifestyle (Asch et al. 2013; Bayer 2008).

Moving forward, the conditions of difficult prevention and moral acceptability of the action per se seem to be met. In pressing conditions of scarcity, choices of prioritization and deprioritization are unavoidable.^5^ Simultaneously, the ability to ensure the alleviation of some compared to the pain, discomfort and eventual loss of eyesight for everyone because of the system’s reluctance to take a relevant decision constitutes a standpoint that unavoidably suggests the moral acceptability of those actions. On that basis, everyone would probably agree that a first-come first-served treatment of the coming patients, with the imprudent ones being more prone to be the first to look for medical care because of their deteriorated eye-health, would express a disrespectful and even discriminatory position towards prudent individuals. A position that overlooks the available information and systematically denies prioritizing the prudent ones and respecting their mindful behaviour and equal moral worth cannot constitute an acceptable action per se. Finally, the deprioritization of the imprudent would not serve as a means to the end of the prudent one’s alleviation. The decision regarding who will be comforted or prioritized is taken simultaneously once and for all the cases, whereas the deprioritization of one patient does not constitute the causal prerequisite for providing adequate treatment to the other. Building on those, and even though the harm minimization condition is not met, the fulfilment of the remaining double effect doctrine’s conditions indicate that we have some responsibility-sensitive reasons to save the least deserving of the danger they face without, however, disrespecting any of the concerned parties.

Consequently, the above conclusion allows me to argue that the aspect of responsibility could be a morally justified element of non-life-threatening triaging decision-making, while everyone is respected, and the greater positive moral value is produced. Under such an approach, everyone is considered worthy of treatment. Yet as a side effect of that consideration, in pressing conditions, some will have to wait longer before being treated or financially contribute to the costs of their treatment.

## Healthy lifestyle and relevant accountability through the lens of a duty for respect

An additional way to demonstrate how a luck egalitarian approach to healthcare provision would manifest instead of undermining respect would be to dive even more deeply into Kantian deontology. Following Kant, human beings constitute rational beings recognized as ends in themselves with dignity (Kant 1998; Dillon 2021). Because of that, we should always ‘act in such a way that we treat humanity, whether in our own person or the person of any other, never simply as a means but always at the same time as an end’ (= The Formula of Humanity) (Kant 1998, p. 4:429). Under that maxim, Kant interpreted another of his core concepts; the one of perfect duties to oneself^6^ and others. Before we move on, it is important to highlight that for Kant any maxim that fails to comply with what these suggest cannot be thought without contradiction, or, in other words, is doomed to inner impossibility (Kant 1998). Simply put, these duties constitute prescriptions of certain kinds of actions from which no exception is permitted due to irrational tendency to a different action or type of behavior. In the context of this paper’s topic, this invites the question: could the preservation of one’s health be regarded as a duty^7^ compatible with the Formula of Humanity? If so, this would suggest the justifiability of personal responsibility’s consideration in the healthcare context as an expression of a respectful mindset both towards oneself and others.

To respond to that question, I will first focus on perfect duties to oneself. Kant invokes the case of a desperate person who is seriously thinking of killing himself to be freed from life’s troubles and difficulties. However, by maintaining his reason, he asks himself whether his maxim—killing oneself out of self-love to escape from a trouble-filled life—treats humanity in his person not only as means but also as an end in itself. It is at once brought out that respect for humanity is completely absent (Kant 1998). But, elaborating on this, one could suggest that the way of killing oneself could differ and be either instantaneous or slow and gradual. Life can be shortened either with a gun or one’s extensive and repeated engagement in bad lifestyle habits, such as heavy smoking, chronic overeating, or excessive drinking. Although not instantaneously, those habits bring death a little bit closer by contributing to the deterioration of health. If this analogy is sound, could the following maxim recognize due respect for humanity and consequently be adopted? Whenever you can engage in extensive bad health habits that fill you with pleasure irrespective of their devastating implications for the quality and length of your life and your dependence on healthcare, you should do so. Even though suicide is not explicitly spelled out, the gradual killing of oneself is implied. If this analogy holds, then this allows us to conclude that, in such cases, humanity is merely used as a means to an end. In our case, this end is the enjoyment of unhealthy lifestyle choices that requires humanity’s sensory perception causing its devastating degradation. Despite not being as strict as our duty to avoid suicide, this perception of one’s unhealthy behaviour and the conclusion suggested above imply forcefully enough our binding perfect duty to protect our health. On this basis, the justifiability of accountability for one’s health status seems more probable. It should now be suggested that a responsibility-sensitive healthcare system could manifest respect for everyone’s equal moral worth without expressing any socially degrading meaning grounded in mental states that overlook everyone’s equal moral worth or fail to recognize due respect to their interests, especially when the focus is on interventions above the basic needs threshold.

For a cognate but self-existent argument that could potentially strengthen my already presented reasoning, let us examine whether it is also plausible to argue that there is a perfect duty to others to preserve our health and be held responsible for failing to respect through these lifestyle choices everyone else’s equal moral worth and right to healthcare. Such a claim would build on the assumption that it is bad to externalize and impose the costs of one’s choices and actions on others. Even though an allegedly reasonable assumption, this might not be universally accepted asking for further defense before we move forward. For that purpose, let me invoke a Dworkinian understanding of what a good life under a just society might look like. Following Dworkin, living well means living in accordance with justice which in its own turn in the social domain means living in conditions where everyone’s efforts to lead a good life are treated as equally important, while mutual respect for everyone’s resources that are properly theirs by not exceeding one’s own fair share at their expense is present (Dworkin 2000). In that spirit, the externalization of the costs of our choices and actions would clearly violate both of these claims, something that would suggest its badness in this and every other context.

Having proved the plausibility of this fundamental assumption, let’s dive into the depth of the argument. According to Kant, for instance, if one was never to pay back borrowed money despite promising to do so, that would contradict the very essence of the concept of promise. Simultaneously, it would fail to treat humanity in others’ person as an end, and consequently, would be unacceptable, suggesting one’s perfect duty to others to keep his promises (Kant 1998). Accordingly, one’s reckless behaviour concerning his health able to question the system’s effectiveness and ability to promote health under conditions of scarcity would contradict others’ right to their fair share in healthcare and life. Consequently, it would be equally unacceptable depicting a disrespectful stance and a problematic mental state regarding others’ equal moral worth and the value of their existence. A reckless behaviour, able to contradict the very essence of the medical system’s mission, and the indifference to everyone’s equal rights and interests in medical care leading sooner or later to an overwhelmed system cannot imply humanity’s handling as both ends and means. Eventually, the reckless exploitation of the provided resources, irrespective of others’ needs and interests, explicitly contradicts any idea of furthering others’ happiness and suggests one’s perfect duty to others not to burden the healthcare system by remaining healthy. A critical reader could claim, however, that this conclusion falls prey to overdemandingness leaving almost no room for moral agency (Axelsen and Nielsen 2020) since it appears to imply that every time one has a fatty meal or smokes a cigarette, he fails his duty to others to preserve his health. To deny this accusation, it sounds reasonable to underline that what I essentially argue for here is that to fail our duty to others we must repeatedly engage in actions for which there is empirical evidence that could likely lead and eventually lead to a considerable deterioration of our health threatening to exceed our fair share in healthcare at others’ expense. Consequently, even though each fatty meal or cigarette might contribute to the eventual outcome, it is the causally relevant aggregated impact of the adopted habit and not an isolated incident that makes us fail our duty, something that entails the reduced demandingness of that.

Finding our way back to the core discussion of this section, adopting a health-responsible behaviour, seems compatible with treating humanity as a means, but most importantly as an end in itself contributing to the promotion of its interests. In contrast, the adoption, even only by some, of a reckless lifestyle would imply that everyone is possibly available for use as a mere means (Tadros 2011), a fact that indicates this stance’s moral unacceptability. Based on these inferences, my argument could be formulated as follows:

- We have a perfect (~ binding) duty to ourselves and others to preserve our health.
- A self-inflicted unhealthy lifestyle may be considered an instance of failing the duties mentioned in a).
- When we fail to abide by the duties suggested under a), we treat some (ourselves or others) as a mere means and disrespect them.
- We have respect-based reasons to seek to moderate, prevent or eliminate instances of such failings.
- A responsibility-sensitive healthcare system could be a mechanism that incentivizes against such failings by treating people as means and ends in themselves and consequently by eliminating disrespect against themselves and others.

Yet, one could still question the plausibility of my step from proving our duties to ourselves and others to preserve our health to justifying on that basis a system that holds us accountable for failing to do so. To suggest the reasonableness of such a step, let me first revisit Dworkin and his positions about the relationship between liberty and equality in the context of a just society and the community’s power to impose that. According to him, ‘(l)iberty is not the freedom to do whatever one wants no matter what, but to do whatever one wants that respects the true rights of others’ (Dworkin 2000, 237). Following that, a community can and should restrict people’s liberty by outlawing any kind of conduct that could potentially violate others’ true rights. ‘It must outlaw theft, for example, to protect people’s rights to security of property’ (Dworkin 2000, 282). In a similar spirit, Tadros (2011) states that when the state’s intervention to effectively coordinate the fulfillment of our relevant moral duties (to others) is required, the state should be arguably justified in imposing coercive measures to facilitate the effective fulfillment of those duties. On those grounds, given our potential failure to comply with our proven duty to preserve our health by expressing disrespect to others’ entitlement to their true rights, a community appears justified and should prevent such conduct through the imposition of coercive measures, i.e. a responsibility-sensitive healthcare system. That inference implies the justifiability of a step from duties to policies.

Summing things up, this argument can explicitly undermine the respect-based critiques’ validity. As already mentioned, this family of objections criticizes luck egalitarianism for an alleged failure to respect, or at least to respect equally for the one or the other reason, all of humanity. My Kantian argumentation around respect seems to be the most suitable tool to remedy this luck egalitarian weakness. By suggesting dignity’s preservation and the subsequent need for respect, even though recklessness for one’s health may call for punishment, rights infringement, or discriminatory exclusion (Dillon 2021), everyone’s equal moral worth is underlined. Respect is depicted as an a priori provision merely because rational beings participate in humanity independently of their earnings (Kant 1998; Dillon 2021). Following these, it seems more reasonable for somebody to argue that respect is preserved rather than eliminated.

Overall, under such a luck egalitarian approach, the reckless ones must bear the costs of their choices as an aftereffect of the respect attributed to their rational volitions’ autonomy and not as an outcome of problematic mental states that fail to recognize due respect for people’s equal moral worth or rate adequately the significance of their interests. Building on that, any related intervention of light abandonment, intrusiveness, and/or differential treatment constitutes either a mere implementation of that respect or an aftermath of one’s failure to abide by the already proven duties and because of that is justified on those grounds. Such a responsibility-sensitive approach concerning healthcare access seems eventually able to constitute the instigator of adopting a better lifestyle contributing in the long-term to the formulation of (self)respect-worthy persons.

## Conclusion

In conclusion, I must highlight once again that both luck egalitarianism and my dominant aim have never been to blame or punish the reckless one for what he has brought about (Albertsen and Knight 2015). On the contrary, the most crucial stake of my reasoning has always been to detect the morally justified role that personal responsibility should play in principle in the bosom of the medical system, especially under critical conditions of scarcity. Devastating actions for one’s health and the existence of reckless agents inescapably seem to be a fact, but how should we respond to that? My argument provides a qualified ‘yes’ to the justifiability of a responsibility-sensitive healthcare system. Simply put, in contrast to what relational egalitarianism claims, this paper argues that there are respect-based reasons in favour of responsibility-sensitive distributive justice. No disrespectful mental state regarding everyone’s equal moral worth seems to justify this reasoning, whereas this responsibility-sensitive approach does not treat anyone as socially unacceptable attributing less respect to their interests than the one they have already attached to those. In addition, deprioritization above the basic needs threshold (~ light abandonment) and the consequent intrusiveness are justified given that they derive from respect for one’s autonomous volition and their failure to comply with their related duties to oneself and others. Nevertheless, we should always be mindful of the dark side of the concept of responsibility. All in all, through the double effect doctrine approach and the underlining of duties of (self-)respect from which we cannot escape, the consideration of personal responsibility is depicted as a depository of human dignity, and not as a form of punishment or disrespectful treatment. Through the lens of this responsibility-sensitive reconsideration of our relations, respect for others and ourselves could flourish, the most cost-effective function of healthcare systems could be achieved, and distributive justice could eventually prevail.

## Data Availability

Not applicable.

## Footnotes

1 The term ‘luck egalitarianism’ was suggested by Elizabeth Anderson (1999), a critic of this viewpoint. However, the term has prevailed and is anymore used by distinctive contemporary advocators of this approach like Knight (2009; 2013), Lippert-Rasmussen (2016), Segall (2010; 2013), and Albertsen (2015; 2016; 2020). I, therefore, also employ it here, even though ‘responsibility-sensitive egalitarianism’ would be an equally fitting term.

2 It should be stated that I discuss luck egalitarianism as a political, and not metaphysical, proposal, something that, for instance, according to Roemer (1993), means that the definition of individual choice and circumstance can potentially derive from the democratically expressed views of the society.

3 Even though both examples could be perceived as building on forward-looking grounds, they constitute cases of backward-looking responsibility-sensitive healthcare policies. Under them, patients are requested to engage in certain lifestyle changes or are excluded from publicly funded treatments due to their preceding health-related acts and behaviors that have considerably contributed to their current need and/or condition.

4 It must be underlined that this does not constitute a universally accepted principle.

5 One of the subtle but core assumptions of my argumentation is that healthcare resources are often scarce, and consequently, decisions of prioritization are required. To justify this general assumption, consider the recent COVID-19 case. During that era, the number of available ventilators and ICU beds was not sufficient for all the hospitalized patients and decisions of prioritization were unavoidably taken. Yet, a critical reader could oppose to my project by arguing that we should focus on how we will ensure the required number of resources, through taxation of the wealthy, for instance, and not on how we could allocate justly the currently available ones. In response to that hypothetical objection, consider the case of organ scarcity. No taxation, irrespective of how excessive this would be, could fight this instance of scarcity back, something that eventually highlights the need for decisions of prioritization. But, in the same vein, consider also the effect of time. In most of the relevant cases, decisions of prioritization are taken under conditions of time pressure and an unexpected emergency. Thus, even though in the long term, increased taxation of the wealthy, or a different measure, could potentially ensure the availability of a sufficient number of resources, in the context of the reality I just sketched out and that we currently live in, we are obliged to engage in discussions and decisions of fair allocation.

6 I am aware of the discussion around the problematic character of duties to oneself that derives from one’s capability to release oneself from the related obligations (Muñoz 2020). Yet, I adopt Ian Carter’s viewpoint arguing that because of continuity and despite temporal distance, liability-responsibility for previews choices is inherited from the current self to the successive one of the same temporally extended agent, an approach that suggests the plausibility of duties to oneself (Carter 2018).

7 By citing examples of duties, similar to the ones mentioned by Kant, at this point, I suggest our perfect duty to oneself and others to protect our health. Yet one must be mindful of the fact that Kant talks about general duties and actions that are regarded as good or bad irrespectively of their consequences. Taking that into account, it seems that my choice to argue in favor of personal responsibility’s consideration invoking a Kantian duty to protect our health would be opposed to my commitment to the condition of the necessary existence of a causal relationship between a reckless action and its devastating outcome so that somebody is held responsible for that (found under the ‘Luck Egalitarian(in health)’ section). In contrast to that, my Kantian argumentation could be regarded as one that implies that we should be held responsible whenever we act recklessly irrespective of our behaviour’s causal relationship with our self-inflicted condition that led us to seek for healthcare. To show that this argument eventually complies with the causal requirement, let me state the following justifications of my choice: Option no.1: Kant focuses on intentions as if he is indifferent to whether they are causally related to a certain problematic outcome. Yet when he presents his examples regarding perfect duties to oneself and others and elaborates on what failing to comply with them would mean, he seems to presuppose that a causal relationship between the action, attributed to the wrong maxim, and the occurrence of the problematic outcome is there. To illustrate that, let me cite here an extract from his text: ‘If he destroys himself in order to escape from a trying condition he makes use of a person merely as a means to maintain a tolerable condition up to the end of life. A human being, however, is not a thing and hence not something that can be used merely as a means, but must in all his actions always be regarded as an end in itself. I cannot, therefore, dispose of a human being in my own person by maiming, damaging or killing him.’ Consequently, in his examples, he assesses an action and whether a person has failed to abide by her duties on the basis of detectable undesirable problematic outcomes and not independently of them. To put it differently, the commission of suicide does not seem to be objectionable per se. In contrast to that, it is objectionable because of its causal relationship with devastating outcomes for the individual, as it is one’s maiming, damage or killing. Based on that, the causal relationship between one’s duty to preserve one’s health and a self-inflicted disease must occur so that he is held responsible for that. Option no.2: Kant focuses on intentions as if he is indifferent to whether they are causally related to a certain problematic outcome. Thus, the described duties are general ones suggesting that we should always take care of our health and be held responsible whenever we fail to do that irrespective of whether this is causally related to our self-inflicted condition or not. Yet, in our society, this cannot really be the case. Each one of us adopts intentionally or not one or more times in her lifetime a bad behavior that would suggest her failure to abide by her duty to preserve her health leading to an over-responsibilization of the medical system which instead of solving some problems of distributive justice, it would create many more questioning the medical system’s current mission. Consequently, so that this is not the case, the causal requirement must apply.
